# Acute in utero exposure to lipopolysaccharide induces inflammation in the pre- and postnatal brain and alters the glial cytoarchitecture in the developing amygdala

**DOI:** 10.1186/s12974-017-0981-8

**Published:** 2017-11-02

**Authors:** Elaine O’Loughlin, Janelle M. P. Pakan, Deniz Yilmazer-Hanke, Kieran W. McDermott

**Affiliations:** 1 Department of Anatomy and Neuroscience, University College Cork, Cork, Ireland; 2 Department of Neurology, Centre of Neurologic Diseases, Brigham and Women’s Hospital, Harvard Medical School, Boston, MA USA; 3 Center for Behavioral Brain Sciences, German Center for Neurodegenerative Diseases, Institute of Cognitive Neurology and Dementia Research, Otto von Guericke University, Magdeburg, Germany; 4 Neurology, Section Clinical Neuroanatomy, Ulm University, Ulm, Germany; 5 Graduate Entry Medical School, University of Limerick, Limerick, Ireland

**Keywords:** Maternal inflammation, Amygdala, Neuroinflammation, Neurodevelopment, Amygdalar development, Inflammatory cytokines, Microglia, Astrogliosis, Lipopolysaccharide (LPS)

## Abstract

**Background:**

Maternal immune activation (MIA) is a risk factor for neurodevelopmental disorders such as autism and schizophrenia, as well as seizure development. The amygdala is a brain region involved in the regulation of emotions, and amygdalar maldevelopment due to infection-induced MIA may lead to amygdala-related disorders. MIA priming of glial cells during development has been linked to abnormalities seen in later life; however, little is known about its effects on amygdalar biochemical and cytoarchitecture integrity.

**Methods:**

Time-mated C57BL6J mice were administered a single intraperitoneal injection of 50 μg/kg lipopolysaccharide (LPS) on embryonic day 12 (E12), and the effects of MIA were examined at prenatal, neonatal, and postnatal developmental stages using immunohistochemistry, real-time quantitative PCR, and stereological quantification of cytoarchitecture changes.

**Results:**

Fetal brain expression of pro-inflammatory cytokines (IL-1β, TNFα, and IL-6) was significantly upregulated at 4 h postinjection (E12) and remained elevated until the day of birth (P0). In offspring from LPS-treated dams, amygdalar expression of pro-inflammatory cytokines was also increased on day 7 (P7) and expression was sustained on day 40 (P40). Toll-like receptor (TLR-2, TLR-4) expression was also upregulated in fetal brains and in the postnatal amygdala in LPS-injected animals. Morphological examination of cells expressing ionized calcium-binding adaptor molecule 1 (Iba-1) and glial fibrillary acidic protein (GFAP) suggested long-term microglial activation and astrogliosis in postnatal amygdalar regions.

**Conclusions:**

Our results showed that LPS-induced MIA at E12 induces a pro-inflammatory cytokine profile in the developing fetal brain that continues up to early adulthood in the amygdala. Inflammation elicited by MIA may activate cells in the fetal brain and lead to alterations in glial (microglia and astrocyte) cells observed in the postnatal amygdala. Moreover, increased pro-inflammatory cytokines and their effects on glial subpopulations may in turn have deleterious consequences for neuronal viability. These MIA-induced changes may predispose offspring to amygdala-related disorders such as heightened anxiety and depression and also neurodevelopmental disorders, such as autism spectrum disorders.

## Background

Compelling evidence suggests that early life challenge, as a result of maternal immune activation (MIA), can have long-lasting, negative effects on neurochemistry, brain excitability, and behavior [4, 5, 22, 43]. Epidemiological studies show that prenatal/neonatal immune activation such as infection, malnutrition, stress, and obstetric complications can increase the susceptibility of individuals to schizophrenia, autism spectrum disorders (ASD), and epilepsy [7, 22, 27]. The literature reveals that infection-induced MIA has profound effects on developing neural circuits with studies suggesting that certain gestational windows are more vulnerable than others (e.g., early versus late pregnancy) to developmentally initiated functional deficits following MIA [22, 29]. Animal models have been developed to study the link between behavioral discrepancies and cellular, molecular, and morphological alterations in the offspring’s brain after prenatal exposure to an immune modulating agents such as polyinosinic:polycytidylic acid (poly I:C) and lipopolysaccharide (LPS) which mimic viral or bacterial infections, respectively [1, 6, 17, 22]. In rodents, early-life exposure to LPS induces the production of pro-inflammatory cytokines such as interleukin (IL)-1β, IL-6, and tumor necrosis alpha (TNFα) both in the periphery and the brain [35]. Additionally, MIA induced with LPS challenge has produced abnormalities such as decreased expression of Reelin (structural marker), increased immunoreactivity for glial fibrillary acidic protein (GFAP), activation of microglial cells, long-term changes in GABAergic function, and decreases in neocortical and hippocampal thickness [12, 15, 26, 32]. Exposure of pregnant dams to other inflammatory agents during gestation also results in deficits that include other hippocampal abnormalities, such as neuronal loss, astrogliosis, and changes in neurotransmitter receptor expression [1, 34, 36]. Several studies have focused on the developing hippocampus, cortex, and mid-brain, but surprisingly, research on MIA-induced amygdalar changes is limited [22]. The amygdala, a limbic structure, is the center for the control of emotions, and disturbances to this component of the limbic system can result in behavioral deficits as seen with anxiety, fear, learning and memory impairments, and also neurodevelopmental disorders such as ASD [20]. Moreover, human MRI studies and in vivo animal studies support the link between developmental disorders and amygdalar involvement [19, 20, 38]. Therefore, the first aim of this study was to investigate the effects of LPS-induced MIA on murine amygdalar development with regard to the cellular, molecular, and cytoarchitectural changes that may occur over key developmental stages.

## Methods

### Animals

All procedures were carried out in accordance with Republic of Ireland Department of Health and Children Licenses. The procedures used in this study had the approval of the Institutional Animal Care and Use Committee and were carried out in accordance with the European Council Directive of November 24, 1986 (86/609/EEC). Pregnant C57BL/6J mice were used and confirmation of a vaginal plug was designated as embryonic day zero (E0) (*n* = 4/group/time point). All pregnant dams were treated at embryonic day 12 (E12) and were randomly assigned to receive a single intraperitoneal (i.p.) injection of either lipopolysaccharide from *Escherichia coli* 026:B6 (LPS; 50 μg/kg; Sigma-Aldrich, Ireland) or 100 μl 0.9% saline vehicle. Male and female offspring were used for all experiments. The first day of birth was termed P0. Developmental stages were divided into prenatal/neonatal (E12, E16, E18, and P0) and postnatal (P7, P14, and P40) stages. All animals were housed on 12-h light/dark cycle (light on 0800 h) at constant temperature of 21 ± 2 °C with food and water available ad libitum*.*

### Tissue preparation

Pregnant dams were anesthetized using isoflurane and decapitated. A midline incision was made into the peritoneal cavity to expose embryos. Control and LPS offspring were sacrificed at seven ages: E12, E16, E18, and P0 (prenatal/neonatal) and P7, P14, and P40 (postnatal). One- and 7-day-old mice were anesthetized by hypothermia, while 14- to 40-day-old mice were anesthetized using isoflurane and promptly decapitated. For molecular analysis, whole brains were removed (E12: 4-h post-, E16: 4-day post-, and E18: 6-day postinjection, and P0: neonatal (first day of birth)) or the amygdalar regions dissected out (P7, P14, and P40), snap-frozen with liquid nitrogen, and stored at − 80 °C until qPCR was carried out. For histological analysis, embryos were dissected out and immersion-fixed in 4% paraformaldehyde (PFA) overnight followed by 30% sucrose (E18 and P0 brains were removed from the skull). Offspring at P7, P14, and P40 were deeply anesthetized and transcardially perfused with 0.05 M PBS followed by 4% PFA in 0.05 M PBS. Brains were removed from the skull and postfixed in 4% PFA overnight followed by 30% sucrose. The litters were split and assigned for molecular or histological assessment.

### Immunohistochemistry (IHC)

Coronal 40-μm-thick sections (one-in-six series) were sectioned using a cryostat (Leica Microsystem GmbH, Wetzlar, Germany). The first and second series of sections were used for immunohistochemistry (IHC). The third series was Nissl-stained with 0.1% Cresyl Violet (CV) to identify structural alterations and neuronal damage. Processed sections were dehydrated, cleared in histolene, and coverslipped using DPX (Sigma-Aldrich, Wicklow, Ireland). The first and second series of sections were processed for immunohistochemistry using specific antisera against glial fibrillary acidic protein (GFAP) (1:2000, ab53554 goat polyclonal GFAP antibody Abcam, Cambridge, UK) and ionized calcium-binding adaptor molecule 1 (Iba-1) (1:1000, ab5076 goat polyclonal Iba-1 antibody Abcam, Cambridge, UK), respectively. Bound antibody was detected using a biotinylated secondary antibody and the avidin-biotin-peroxidase complex (ABC) method (Vector laboratories, Peterborough, UK). Immunohistochemical staining was visualized using 3′,3′-diaminobenzidine (Sigma Aldrich, Wicklow, Ireland) as a chromogen.

### Microglia (Iba-1) cell counting and astroglial (GFAP) scoring

The amygdala region was delineated in postnatal animals using the mouse atlas. Iba-1 staining was used to examine the morphology of microglial cells. For each animal case, four sections were quantified and the number of total number of Iba-1-positive cells was counted with aid of ImageJ software. Iba-1-positive cells were divided into activated and resting and calculated as a percentage of total microglia present in that region. Iba-1-positive cells were counted in the postnatal amygdala and subdivided into ramified versus amoeboid-like morphology [40]. The quantification is displayed as total (%) of cells counted in the region. GFAP staining was analyzed as an indication of astrogliosis and inflammation in the amygdala in MIA offspring in comparison to saline-treated controls. GFAP-positive immunostaining per region was scored (*n* = 4 per treatment/group) with slight modifications according to O’Loughlin et al. [30] by performing background correction with the aid of ImageJ software. Density of GFAP-positive cells and variation in the staining intensity (somata and cellular processes) were included into scoring in a blinded manner as − = none (0% stained), + = very few cells (< 10% of area stained), ++ = few cells (10–25% of area stained), +++ = moderate number of cells (25–50% of area stained), and ++++ = numerous cells (> 50% stained).

### Quantification of postnatal amygdalar volumes and neurons

Volumes of postnatal amygdalar regions were assessed according to the Cavalieri principle [16]. Amygdalar nuclei were delineated in Nissl-stained sections using a mouse atlas [13]. Consecutive sections (section sampling fraction (ssf) 1:6, sections 120 μm apart from each other) were sampled in saline- and LPS-injected offspring at three ages: P7, P14, and P40. Volumes were calculated using the formula: *V = ∑P*_*i*_ *· a*(*p*) *·d*, where *P*_i_ is the sum of hit point counts in all sections, *a*(*p*) is the area associated with each point, and *d* is the section spacing. In the amygdala, the volumes of the lateral (LA), basolateral (BLA), and central (Ce) nuclei were measured in consecutive sections, commencing at Bregma − 0.70 to − 2.30 mm according to the mouse atlas [13]. Total numbers of neurons were estimated in Nissl-stained amygdalar nuclei using the optical dissector method [16]. Neurons were distinguished from astrocytes and oligodendrocytes based on cell and nuclear size, amount of nuclear heterochromatin, and organization of nucleolus [9]. Consecutive sections (1 in 6 series; 120 μm apart) throughout the rostro-caudal extent of the amygdala were used in saline- and LPS-injected offspring at three ages: P7, P14, and P40, (Bregma − 0.70 to − 2.30 mm) [13]. An Olympus BX 40 microscope (×100 objective) was used to count the neurons. An unbiased counting frame with inclusion and exclusion lines was superimposed, and neurons that lay between the boundaries were counted. The focal plane was moved down the *z*-axis using a digimatic micrometer that was attached to the stage of the microscope to measure the *z*-axis depth. The following equation was used: *E*(*N*) *= Nv X Vref*, as previously described in detail elsewhere [41].

### RNA extraction, cDNA synthesis, and qPCR assay

Total RNA was extracted from brain/amygdala samples using Qiagen RNeasy Plus Lipid mini kit as per manufacturer’s guidelines (Qiagen, Crawley, UK). Next, cDNA was generated from total RNA using a High Capacity Reverse Transcriptase Kit (Applied Biosystems, Warrington, UK). All real-time quantitative PCR were performed using StepOne™ Real-Time PCR system (Applied Biosystems) with TaqMan® Gene expression Master Mix (Applied Biosystems) and the specific TaqMan® Gene Expression Assay for the following targets: *IL-β* (Mm00434228_m1), *TNFα* (Mm00443258_m1), *IL-6 (*Mm00446190_m1), *TLR-2* (Mm00442346_m1), *TLR-4* (Mm00445273_m1), *COX-2* (Mm00478374_m1), *iNOS* (Mm00440502_m1), *CD36* (Mm00432403_m1), and *CCL2* (Mm00441242_m1) (Applied Biosystems). All samples were run in triplicate. The mRNA expression levels between samples were normalized using β-Actin (Mm02619580_g1) endogenous control (VIC dye labeled, Applied Biosystems™). The quantification of mRNA expression in the LPS treatment groups relative to the saline control (fold change) was done using the 2^−∆∆C^_T_ method [39].

### Statistics

Statistical analyses were carried out using GraphPad Prism software version 7. Data were represented as mean values ± the standard error of the mean (SEM). For histology quantification, Student’s *t* test was used to compare parametric data of two treatment groups. Gene expression profiles were analyzed with one-way ANOVA followed by Tukey’s multiple-comparison post hoc test. Differences were considered to be significant only for ***p < 0.001, **p < 0.01, and *p < 0.05. There were four pregnant mice (*n* = 4) in each treatment group/time point.

## Results

### Bacterial induced MIA increases the production of inflammatory cytokines in the prenatal brain and postnatal amygdala

**Fig. 1:**
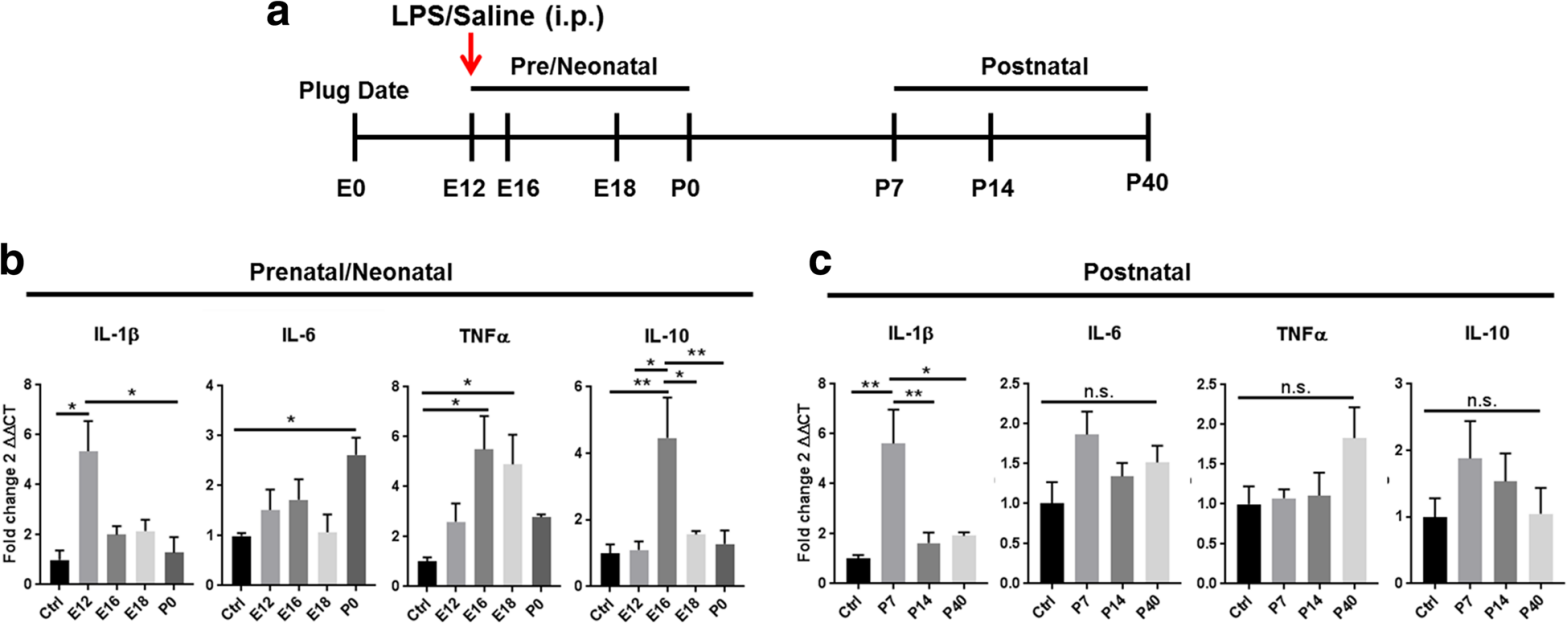
Maternal LPS induced inflammatory response in prenatal/neonatal brains and postnatal amygdala of challenged offspring. Maternal immune activation induces elevated circulating cytokines in the prenatal/neonatal and postnatal CNS (**b**, **c**). **a** Schematic representation of experimental setup. **b**, **c** Gene expression was normalized against β-actin via ∆Ct. Results are mean normalized to expression ± SEM (*n* = 4/treatment group/time point). Significant difference denoted as *p < 0.05, **p < 0.01, and ***p < 0.001 in post hoc multiple comparisons performed using Tukey’s test following one-way ANOVA

We first investigated the effect MIA on inflammatory cytokine expression at different stages of development. The amygdala nuclei originate from different domains of the embryonic brain maturating at different developmental stages [25], and the size of the amygdala was too small to be dissected out reliably in the pre- and neonatal brain. Therefore, we divided the developmental ages into two groups (prenatal/neonatal and postnatal). Whole brain lysates were analyzed at E12 (4-h postinjection), E16, E18, and P0 in prenatal/neonatal stages, and the amygdala was dissected under the microscope at postnatal stages P7, P14, and P40 (Fig. 1a). One-way ANOVA for the main factor age showed a significant effect for *IL-1β* (*F*(_4, 15_) = 5.97, *p* = 0.010, *TNFα* (*F*(_4, 15_) = 4.93, *p* = 0.0186), *IL-6* (*F*(_4, 15_) = 3.178, *p* = 0.05), and *IL-10* (*F*(_4, 15_) = 7.713, *p* = 0.0042) in the prenatal/neonatal development stages. Likewise, a significant age effect was observed postnatally for *IL-1β* (*F*(_3, 12_) = 8.42, *p* = 0.0028), but no significant differences were recorded for *IL-6* (*F*(_3, 12_) = 2.27, *p* = 0.1325), *TNFα* (*F*(_3, 12_) = 2.013 *p* = 0.1660), or *IL-10* (*F*(_3, 12_) = 0.982, *p* = 0.4337). Data showed there was a rapid response to MIA-LPS with mRNA induction of several pro-inflammatory genes including IL-1β and TNFα. Specifically, MIA significantly increased mRNA expression of *IL-1β* at E12 by sixfold as indicated by post hoc analyses (Fig. 1b; *p = 0.011). Expression levels were also significantly higher at E12 in comparison to P0 (Fig. 1b; *p = 0.011). In the postnatal amygdala, *IL-1β* was increased approximately sixfold at P7 with levels decreasing but remaining significantly elevated at P40 (Fig. 1c; **p = 0.0032). *IL-1β* was also higher at P7 in comparisons to P14 (**p = 0.0095) and P40 (*p = 0.0156). *IL-6*, an important neuro-immune regulator, was increased about threefold after MIA on the first postnatal day P0 (Fig. 1b; *p = 0.05). In the postnatal amygdala, *IL-6* was elevated across all ages (P7 to P40), which however did not reach significance (Fig. 1c). *TNFα*, another pro-inflammatory cytokine, was elevated across prenatal ages with significant increases at E16 (*p = 0.019) and at E18 (*p = 0.044) in comparison to saline-treated controls, but was not significantly increased in the postnatal amygdala (Fig. 1b, c). *IL-10* is a known anti-inflammatory cytokine, and its expression was significantly increased at E16 in the prenatal brain in comparison to saline-treated controls (Fig. 1b; **p = 0.003). *IL-10* mRNA levels were higher at E16 in comparison to E12 (*p = 0.0103), E18 (*p = 0.014), and P0 (**p = 0.0057) but remained unaffected at postnatal ages as indicated by post hoc analyses. Taken together, the data shows that offspring from MIA-induced dams had an elevated inflammatory response in the CNS from 4 h postinjection to early adolescence at P40.

### Acute maternal exposure to LPS induces increased expression of inflammatory mediators

**Fig. 2:**
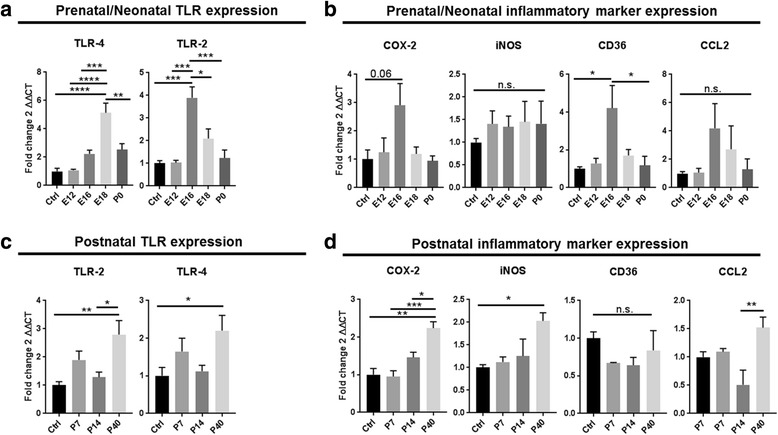
MIA-induced an inflammatory milieu in prenatal/neonatal brain (**a**, **b**) and postnatal amygdala (**c**, **d**) in offspring. Expression of inflammatory markers and toll-like receptors (TLRs) in prenatal/neonatal brains and postnatal amygdala (**a**–**d**). TLR-4 and TLR-2 mRNA expression profiles were elevated in the developing brain and remained significantly higher up to 40 days post insult. Gene expression was normalized against β-actin via ∆Ct. Results are mean normalized to expression ± SEM (*n* = 4/treatment group/time point). Significant difference denoted as *p < 0.05, **p < 0.01, and ***p < 0.001 in post hoc multiple comparisons performed using Tukey’s test following one-way ANOVA

Toll-like receptors (TLRs) are known to mediate immune responses and induce inflammatory cascades. TLR-4 is thought to mediate bacterial responses in such that LPS is a ligand of this receptor. TLR-2 is also involved in LPS-induced TLR-4 signaling and thus inflammation [21]. Therefore, we investigated the effect of LPS-induced MIA on TLR expression across stages of amygdalar development (Fig. 2a, c). One-way ANOVA for the main factor age showed a significant effect for *TLR-2* (*F*(_4, 15_) = 13.24, *p* < 0.0001) and *TLR-4* (*F*(_4, 15_) = 18.3, *p* < 0.0001) in the prenatal/neonatal development stages. Likewise, a significant age effect was observed postnatally for *TLR-2* (*F*(_3, 12_) = 6.45, *p* = 0.0075) and *TLR-4* (*F*(_3, 12_) = 3.354, *p* = 0.05). In post hoc analyses, *TLR-2* was significantly increased at E16 relative to saline-treated controls (***p = 0.0002) and to LPS-treated offspring at E12 (***p = 0.0002), E18 (*p = 0.013), and P0 (***p = 0.0004). In the postnatal amygdala, *TLR-2* expression was significantly increased at P40 in comparisons to saline-treated controls (**p = 0.007) and to P14 (*p = 0.0232). *TLR-4* mRNA expression was significantly increased at E18 in comparison to saline treatment (****p < 0.0001) as well as to E12 (****p < 0.0001), E16 (***p = 0.0008), and P0 (**p = 0.0022), the prenatal/neonatal stages. In the postnatal amygdala, *TLR-4* expression was increased at P40 in comparison to saline-treated controls and was unaffected over other postnatal ages examined (*p = 0.050). One-way ANOVA for the main factor age showed a significant effect for *COX-2* (*F*(_4, 15_) = 3.263, *p* = 0.0464) and *CD36* (*F*(_4, 15_) = 4.257, *p* = 0.0203) but did not record significant differences for *iNOS* (*F*(_4, 15_) = 0.279, *p* = 0.8862) or *CCL2* (*F*(_4, 15_) = 1.403, *p* = 0.2872). Likewise, a significant age effect was observed postnatally for *COX-2* (*F*(_3, 11_) = 12.74*, p* = 0.0007), *iNOS* (*F*(_3, 11_) = 3.676, *p* = 0.0470), and *CCL2* (*F*(_3, 12_) = 5.909, *p* = 0.0103), but no significant differences were noted for *CD36* expression (*F*(_3, 12_) = 1.277, *p* = 0.3265). In post hoc analyses, mRNA expression of the prostaglandin-synthetizing enzyme cyclooxygenase *COX-2* was significantly increased at P40 in comparison to saline-treated controls (Fig. 2b, d **p = 0.0012) and to offspring treated with LPS at P7 (***p = 0.0009) and P14 (*p = 0.0281). *CD36*, a scavenger receptor expressed by activated microglia/macrophages in the CNS, is implicated in inflammatory responses [37]*. CD36* mRNA expression was significantly increased at E16 in comparison to saline-treated controls (*p = 0.024) and higher at E16 versus P0 (*p = 0.035) but remained unaffected in the postnatal amygdala (Fig. 2b, d). Chemokine (c-c motif) ligand 2 (*CCL2*) was increased but did not reach significance in the prenatal/neonatal ages studied (Fig. 2b, d). Post hoc analyses showed that *CCL2* expression was significantly elevated at P40 in comparison to P14 (Fig. 2d, **p = 0.006). The inducible form of nitric oxide (NO) synthase, *iNOS*, was significantly upregulated in the postnatal amygdala at P40 in comparison to saline-treated controls (Fig. 2d *p = 0.042). Together with the enhanced mRNA levels observed for *COX-2*, this elevation in *iNOS* mRNA expression in MIA offspring may indicate a heightened inflammatory response in the postnatal amygdala (Fig. 2b, d).

### Astrocyte and microglial environment is altered in the developing amygdala after MIA

**Fig. 3:**
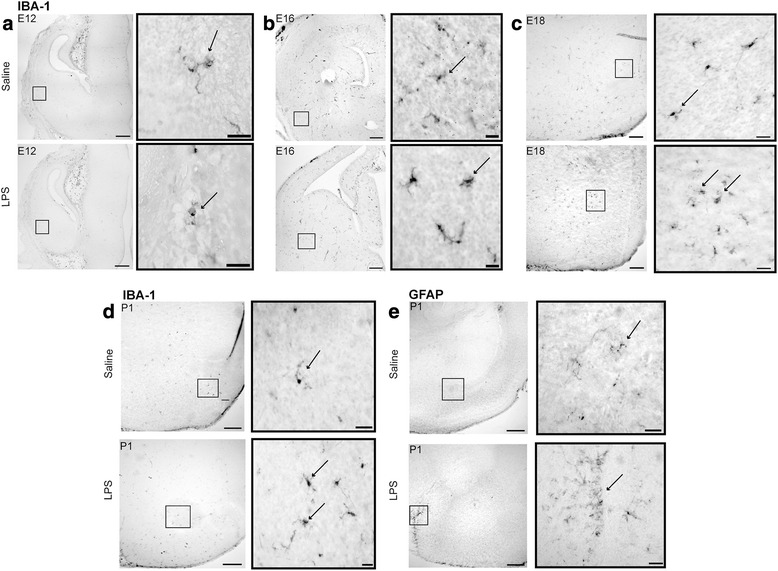
MIA induced changes in microglial morphology in the developing amygdala. Iba-1 immunoreactivity (ir) at **a** E12, **b** E16, **c** E18, and **d** P1. GFAP expression was first present at P0 with no GFAP expression preceding this time point (**e**). Iba-1 positively labeled microglia exhibited ramified and amoeboid morphologies from E12 to P0. In LPS-treated groups, microglial morphology was predominantly amoeboid with GFAP immunoreactivity being dense along the external capsule of the amygdala. Astrocytes were hypertrophic in the LPS-treated group at P1. Scale bar = 200 μm and inset images scale bar = 50 μm

**Fig. 4:**
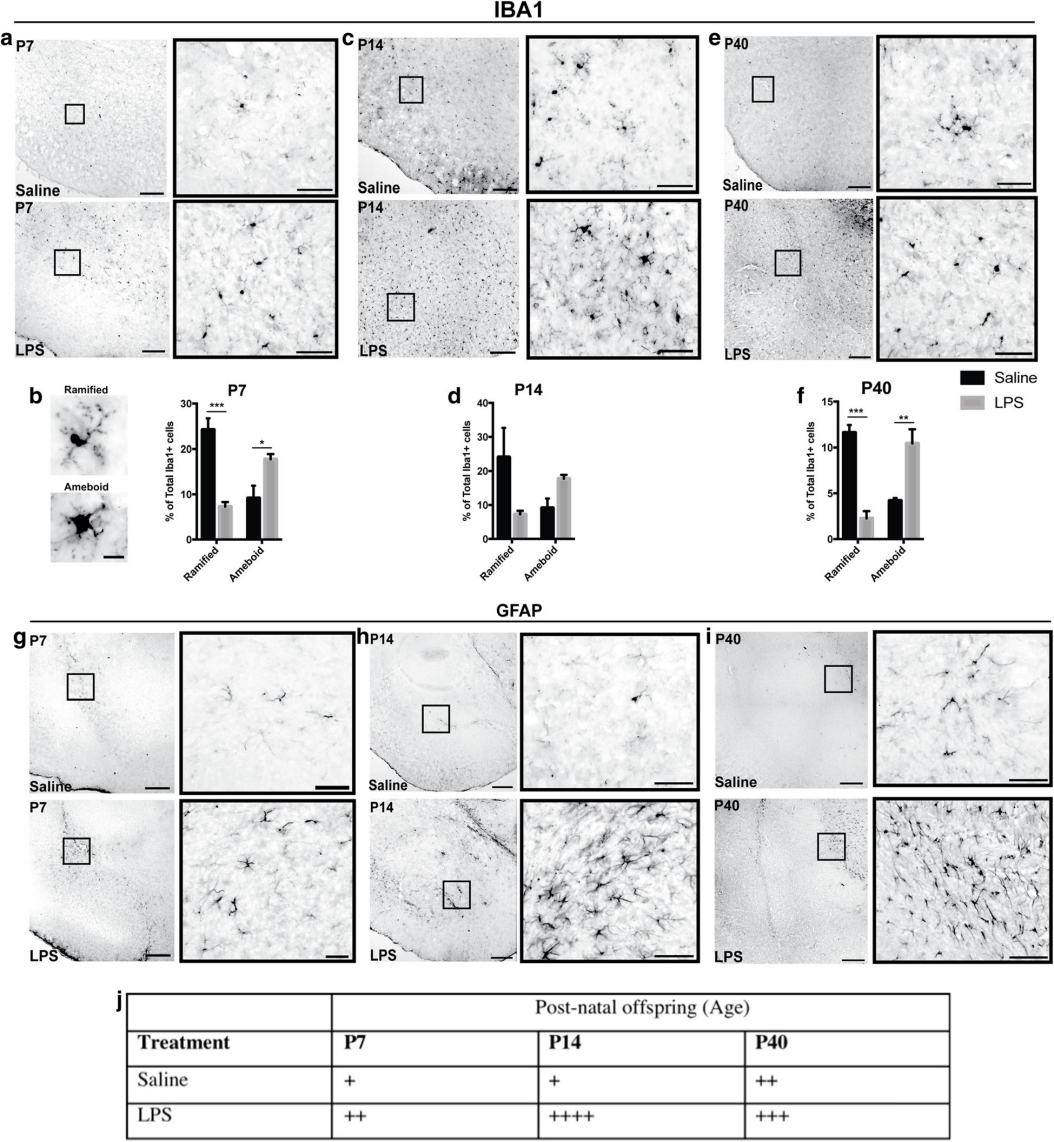
Increased microgliosis and astrogliosis in the postnatal amygdala after MIA. Iba-1 immunoreactivity in the saline and LPS-treated animals also displayed distinct differences revealing clear changes in cell morphology. At higher magnification (insets of panels **a**, **c**, and **e**), Iba-1-positive cells display ramified morphologies in saline-treated animals, whereas in LPS animals, they are more widely distributed and exhibit amoeboid morphology (large cell body with thick stunted processes). Representative images (**b**) of Iba-1 positive microglia that were quantified; morphologies of microglia: top panel shows a ramified microglia with long processes; lower panel shows microglia with thick cell body, stunted processes and amoeboid morphology (**b**). Counts of Iba-1-positive microglia showed higher percentage of amoeboid-like microglia at P7 and P40 in LPS-treated groups in comparison to saline-treated controls with no significant differences seen at P14 (**b**, **d** and **f**). GFAP immunoreactivity was sparse in saline animals especially at earlier postnatal ages; however, it increased considerably in the LPS-treated animals (**g**, **h** and **i**). Astrocyte territories overlap and exhibit cellular hypertrophy indicative of astrogliosis at P14 (**h**, bottom inset) and P40 (**i**, bottom inset). Semi-quantitative scoring of GFAP staining intensities showed that the amygdala had higher GFAP score at P14 in the LPS-treated groups (**j**). Across the ages examined, LPS-treated groups had relatively higher GFAP staining intensities in comparison to saline control animals (**j**). Density of GFAP-positive cells and variation in the staining intensity (somata and cellular processes) were included into scoring in a blinded manner as − = none (0% stained), + = very few cells (< 10% of area stained), ++ = few cells (10–25% of area stained), +++ = moderate number of cells (25–50% of area stained), and ++++ = numerous cells (> 50% stained) (**j**). Scale bar = 200 μm with inset images scale bar = 50 μm. Significant difference denoted as *p < 0.05, **p < 0.01, and ***p < 0.001

We next sought to determine if there were morphological alterations in microglia and astrocytes over the same time course after MIA induction in the prenatal brain (E12-P0) (Fig. 4) and postnatal amygdala (P7-P40) (Figs. 3 and 4). Morphological analysis of Iba-1 immunoreactivity (ir) revealed that microglia had larger cell bodies and thicker shortened processes consistent with a de-ramified morphological profile in LPS groups. During neurodevelopment, microglia have an amoeboid morphology consistent with their phagocytic processes and synaptic pruning; however, after MIA insult, these cells exhibit an amoeboid phenotype with an activated status. This was evident in the postnatal ages, as semi-quantitative analysis showed that the predominant microglial phenotype was amoeboid with an “activated” phenotype in offspring from MIA dams. At P7, there was a significant reduction in ramified-like “resting” microglia (***p < 0.001), whereas microglial cells were predominantly amoeboid after MIA (*p < 0.05). This trend was also significantly different at P40 and more robust (Fig. 4b, f; *p < 0.05, ***p < 0.001). GFAP-ir, a marker for astrocytes that is upregulated in inflammation and cell damage, showed moderate astrogliosis in the amygdalar region with bushy-like morphology and overlapping processes in MIA offspring in comparison to saline-treated controls (Fig. 4). In addition, GFAP-ir was more intense in the amygdalar regions with increasing age and treatment. Labeled astrocytes indicative of astrogliosis were prominent along the external capsule and also in the amygdalar region in MIA offspring, in particular at P14 (Fig. 4j). Semi-quantitative scoring of GFAP-ir revealed the highest GFAP staining at P14 in MIA offspring in comparison to controls (Fig. 4j).

### MIA induced cytoarchitectural changes in the central nucleus at P14

**Fig. 5:**
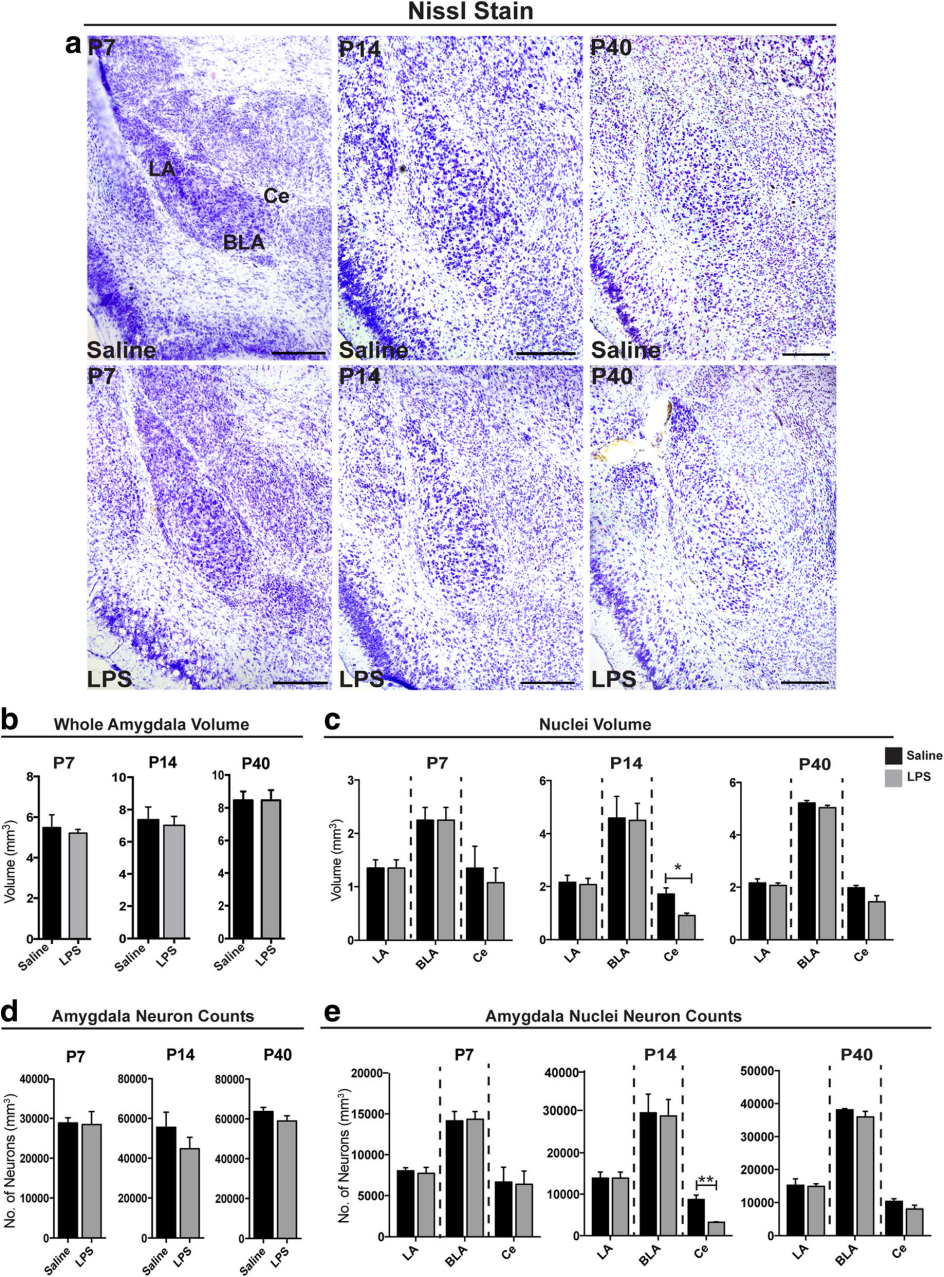
MIA induced cytoarchitectural changes in the postnatal amygdala. Nissl staining was carried out to examine the structural alterations in the postnatal amygdala after MIA (**a**). In addition, stereological analyses were carried out to measure volume differences and cell counts in the developing amygdala in LPS and saline-treated control pups (**b**-**e**). Analyses revealed that the amygdala at P14 is most vulnerable to neuron and volume loss, in particular the central nuclei (**b**-**e**). A decrease in volume and also cell number was observed in the Ce at P14 (**c**, **e**). However, P7 and P40 remained unaffected with respect to LPS or saline treatment (**b**-**e**). Comparisons were carried out between each treatment with respect to treatment using the Student t test. Significant difference denoted as *p < 0.05, **p < 0.01, and ***p < 0.001

After examining the cytokine profiles in the CNS after MIA, next we wanted to investigate the cytoarchitecture of the postnatal amygdala (Fig. 5). It is known that increased cytokine expression may have negative effects on brain structure [28]. Here, we investigated the cytoarchitecture of the postnatal amygdala after E12 maternal injection at P7, P14, and P40 in both treatment groups with the aid of Nissl stain and classification of cell types according to [9] (Fig. 5). Volumetric analysis revealed that the central nucleus (*Ce*) was significantly smaller at P14 after maternal LPS treatment (*p < 0.05). Moreover, total cell counts using Nissl stain showed a significantly lower number of the total neuron population (**p < 0.01).

## Discussion

Inflammation during pregnancy (MIA) can affect several aspects of neurodevelopment, with disturbances that may initiate a series of events eventually interrupting neuronal and glial functions. MIA can induce a “cytokine storm” in the fetal brain, which has been shown to have negative effects on brain structure, both biochemically and structurally. Studies inducing MIA via LPS challenge have shown that this results in increased microglial activation with alterations in morphology from “resting” ramified to an “activated” amoeboid cell type [5]. These changes may lead to downstream effects on the cytoarchitecture of the amygdala as seen at P14, a period known to be important for brain maturation [11]. The present in vivo study demonstrates that an acute maternal LPS injection at E12 upregulates pro-inflammatory cytokines and toll-like receptors in the fetal CNS that subsequently increases glial cell immunoreactivity, possibly leading to a developmental delay and/or cell loss in the postnatal amygdala.

TLRs are expressed on most immune-cell types, in particular on microglia, which increase TLR expression in response to inflammatory stimuli. TLR-4 is a known receptor for LPS, and here, after MIA induction, *TLR-4* was upregulated in fetal CNS tissue and remained upregulated in late postnatal ages suggestive of glial activation. Moreover, *TLR-2* that is also known to be elevated after inflammation was upregulated at prenatal/neonatal and postnatal developmental ages. In addition, *COX-2*, *iNOS*, and *CCL2* were also significantly upregulated at P40 which may further suggest prolonged glial “activation” state. Taken together, an acute bacterial challenge during a critical window of amygdalar development maybe sufficient to induce inflammatory mediators and TLRs in the developing CNS that may persist and affect the later development of the amygdala. However, it should be taken into consideration that other CNS cells such as astrocytes and/or infiltrating immune cells (monocytes, macrophages, etc.) can also produce pro-inflammatory cytokines. During development, the BBB is not fully formed, and infiltration from the periphery is highly likely. While our study examined gene expression in whole tissue lysates, isolation of microglial cells using other technologies such as flow cytometry might help characterizing immune cells involved in MIA effects in future studies.

Our data suggests a particular effect at P14 on the central nucleus (Ce) of the amygdala that had lower neuronal numbers and corresponding volume reduction compared to saline-treated controls. Astrogliosis, which is an indicator of cell damage and inflammation, was most evident in the amygdala at the same developmental stage. The reduction in Ce volume and cell numbers in the LPS group at P14 may indicate a developmental delay at this time point, considering that amygdalar development and growth continues postnatally [14], because both the volume and cell numbers in the Ce reached adult levels as in saline-treated offspring at P40. Studies in rodents have shown that postnatal day 14 (P14) is a crucial period for brain maturation [36] and synaptic pruning during this postnatal development is known to play a vital role [31]. Synaptic pruning is particularly efficient at P14 in mice, and alterations to the microglial functions induced by an inflammatory event during this time or earlier may contribute to synaptic abnormalities seen in some neurodevelopmental disorders [42]. Adolescence is an important time of neurobehavioral maturation during which limbic structures such as the hippocampus, amygdala, and prefrontal cortex undergo maturation [8, 23]. It has been shown that alteration to the microglial environment during early life impairs synaptic elimination and negatively impacts on functional behaviors [42]. After maternal LPS, fetal brain *IL-1β* mRNA gene expression levels were significantly elevated as soon as 4 h postinjection. The elevated pro-inflammatory milieu early in development may “activate” glial cells such that in later postnatal stages, they display activated phenotypes as indicated by increased *CCL2, COX-2*, and *iNOS* with TLR-2 and TLR-4 expression gene expression and also increased Iba-1 and GFAP immunoreactivity and morphological changes. This is in agreement with studies showing that prenatal exposure to LPS induces elevated *IL-1β* expression and is associated with postnatal cell death and gliosis [33].

Interestingly, CD36-TLR-4 signaling is implicated in microglial inflammatory response that promotes neurotoxicity [37]. Our data showed that CD36 is elevated at prenatal stages, which coincides with increased expression of TLR-4. This elevated CD36/TLR-4 expression prenatally may enhance the inflammatory profile and thus microglial priming in the developing brains after MIA. The transient upregulation of the anti-inflammatory cytokine *IL-10* at E16 might have contributed to the temporary decline in *TLRs* and some pro-inflammatory mediators in neonatal MIA offspring and at early postnatal stages, but postnatal *IL-10* levels remained unaffected. While analyses of further anti-inflammatory markers may allow a more comprehensive picture of MIA-induced signaling, the current results with upregulation of *IL-1β* at P7 and the pro-inflammatory molecular profile found at P40 support the notion that a single prenatal LPS injection can cause long-term alterations in the forebrain including upregulation of TLRs, potentially permanently altering the brain’s responsivity to infectious agents and inflammation. Elevated expression of inflammatory molecules may combine together to have a contributing negative effect on neurons in the postnatal amygdala, e.g., by impairing neurogenesis, inhibiting migration and differentiation, and/or inducing cell loss. However, the precise mechanisms by which toxin-induced MIA induces fetal brain inflammation and function remain elusive. It has been demonstrated that it is not the toxin itself that directly induces the effects but the soluble mediators (cytokines, chemokines, reactive species) produced via downstream signaling cascades [2]. Therefore, other glial cells and infiltrating macrophages and/or monocytes should not be ignored. *CCL2* is expressed by other myeloid cells, such as monocytes, and acts as a chemoattractant; in particular, it is involved in recruiting cells to sites of injury. Increased expression of *CCL2*, as seen in our data, may induce recruitment of other myeloid cell populations to the CNS in response to neuroinflammation. Alternatively, activated astrocytes and microglial cells may release CCL2 after MIA insult. The involvement and mechanisms of recruitment of different inflammatory cells to the developing CNS should be explored in future studies. Furthermore, MIA may influence immune mediators at the protein level through various mechanisms, e.g., miRNAs can alter the expression of TAB3 downstream of *IL-1β* signaling after MIA induction [18] or posttranslational modifications may take place.

In mice, a disturbance in cytokine signaling via receptor blockade or genetic deletion can produce alterations in ASD-like phenotypes including memory deficits and social withdrawal behaviors [3]. Furthermore, prenatal MIA on E9-E12 enhances anxiety- and depressive-like behavior in adult offspring [10, 24]. The central nucleus is the major output station of amygdalar projections, and as our data shows, the Ce structure is compromised after MIA, which could have negative effects for neurobehavioral outcomes. For example, MIA-induced changes in the Ce could alter visceral and motor responses to anxiogenic and emotionally arousing stimuli. Because the Ce is a major regulator of the HPA axis, alterations in the cell types and function of the Ce may also affect inflammation by altering glucocorticoid hormone function [20]. Thus, despite comparable neuronal cell numbers between MIA and saline groups at P40, the function of the Ce may be permanently altered after MIA exposure, because specific subsets of peptidergic Ce projection neurons innervating certain hypothalamic and brainstem regions may be affected. While our study examined the cytoarchitectural effects of MIA challenge over development, further studies using our model may be beneficial for correlating the structural changes in the amygdala with behavioral outcomes of this insult, e.g., ASD-like phenotypes.

## Conclusion

In conclusion, there is a complex interplay between the immune and neural signaling in the developing brain, and when an insult or disturbance such as MIA occurs, this may negatively affect the cytoarchitecture and function of the amygdala. Our model of maternal inflammation induced an elevated cytokine and TLR response in the fetal brain, which is associated with moderate astrogliosis, activated microglia, and cytoarchitectural changes in the postnatal amygdala. Our data suggests that the developing amygdala may be particularly sensitive to maternal stimuli that may influence genetically determined developmental processes.

## References

[1] Arsenault D, St-Amour I, Cisbani G, Rousseau LS, Cicchetti F (2014). The different effects of LPS and poly I:C prenatal immune challenges on the behavior, development and inflammatory responses in pregnant mice and their offspring. Brain Behav Immun.

[2] Ashdown H, Dumont Y, Ng M, Poole S, Boksa P, Luheshi GN (2006). The role of cytokines in mediating effects of prenatal infection on the fetus: implications for schizophrenia. Mol Psychiatry.

[3] Ashwood P, Krakowiak P, Hertz-Picciotto I, Hansen R, Pessah I, Van de Water J (2011). Elevated plasma cytokines in autism spectrum disorders provide evidence of immune dysfunction and are associated with impaired behavioral outcome. Brain Behav Immun.

[4] Bauman MD, Iosif AM, Smith SE, Bregere C, Amaral DG, Patterson PH (2014). Activation of the maternal immune system during pregnancy alters behavioral development of rhesus monkey offspring. Biol Psychiatry.

[5] Bilbo SD, Schwarz JM (2009). Early-life programming of later-life brain and behavior: a critical role for the immune system. Front Behav Neurosci.

[6] Boksa P (2010). Effects of prenatal infection on brain development and behavior: a review of findings from animal models. Brain Behav Immun.

[7] Brown AS (2012). Epidemiologic studies of exposure to prenatal infection and risk of schizophrenia and autism. Dev Neurobiol.

[8] Casey BJ, Jones RM, Hare TA (2008). The adolescent brain. Ann N Y Acad Sci.

[9] Chareyron LJ, Banta Lavenex P, Amaral DG, Lavenex P (2011). Stereological analysis of the rat and monkey amygdala. J Comp Neurol.

[10] Depino AM (2015). Early prenatal exposure to LPS results in anxiety- and depression-related behaviors in adulthood. Neuroscience.

[11] Dinel AL, Joffre C, Trifilieff P, Aubert A, Foury A, Le Ruyet P, Laye S (2014). Inflammation early in life is a vulnerability factor for emotional behavior at adolescence and for lipopolysaccharide-induced spatial memory and neurogenesis alteration at adulthood. J Neuroinflammation.

[12] Fatemi SH, Emamian ES, Sidwell RW, Kist DA, Stary JM, Earle JA, Thuras P (2002). Human influenza viral infection in utero alters glial fibrillary acidic protein immunoreactivity in the developing brains of neonatal mice. Mol Psychiatry.

[13] Franklin KBJ, Paxinos G (2008). The mouse brain in stereotaxic coordinates.

[14] García-López M, Abellán A, Legaz I, Rubenstein JL, Puelles L, Medina L. Histogenetic compartments of the mouse centromedial and extended amygdala based on gene expression patterns during development. J Comp Neurol 2008;506(1):46-74. PubMed PMID: 17990271; PubMed Central PMCID: PMC4916653. 10.1002/cne.21524 PMC4916653 17990271

[15] Ghiani CA, Mattan NS, Nobuta H, Malvar JS, Boles J, Ross MG, Waschek JA, Carpenter EM, Fisher RS, de Vellis J (2011) Early effects of lipopolysaccharide-induced inflammation on foetal brain development in rat. ASN Neuro. 2011;3(4):10.1042/AN20110027. 10.1042/AN20110027 PMC3218569 22007738

[16] Gundersen HJ, Jensen EB (1987). The efficiency of systematic sampling in stereology and its prediction. J Microsc.

[17] Harvey L, Boksa P (2012). Prenatal and postnatal animal models of immune activation: relevance to a range of neurodevelopmental disorders. Dev Neurobiol.

[18] Hu E, Ding L, Miao H, Liu F, Liu D, Dou H, Hou Y. MiR-30a attenuates immunosuppressive functions of IL-1β-elicited mesenchymal stem cells via targeting TAB3. FEBS Lett. 2015;589(24 Pt B):3899–907. doi:10.1016/j.febslet.2015.11.001. Epub 2015 Nov 7. PubMed PMID: 26555189. 10.1016/j.febslet.2015.11.001 26555189

[19] Ishikawa J, Nishimura R, Ishikawa A (2015). Early-life stress induces anxiety-like behaviors and activity imbalances in the medial prefrontal cortex and amygdala in adult rats. Eur J Neurosci.

[20] Janak PH, Tye KM (2015). From circuits to behaviour in the amygdala. Nature.

[21] Kawai T, Akira S (2011). Toll-like receptors and their crosstalk with other innate receptors in infection and immunity. Immunity.

[22] Knuesel I, Chicha L, Britschgi M, Schobel SA, Bodmer M, Hellings JA, Toovey S, Prinssen EP (2014). Maternal immune activation and abnormal brain development across CNS disorders. Nat Rev Neurol.

[23] Konrad K, Firk C, Uhlhaas PJ (2013). Brain development during adolescence: neuroscientific insights into this developmental period. Dtsch Arztebl Int.

[24] Majidi-Zolbanin J, Doosti M-H, Kosari-Nasab M, Salari A-A (2015). Prenatal maternal immune activation increases anxiety- and depressive-like behaviors in offspring with experimental autoimmune encephalomyelitis. Neuroscience..

[25] Medina L, Bupesh M, Abellán A. Contribution of genoarchitecture to understanding forebrain evolution and development, with particular emphasis on the amygdala. Brain Behav Evol. 2011;78(3):216–36. doi:10.1159/000330056. Epub 2011 Aug 23. Review. PubMed PMID: 21860224. 10.1159/000330056 21860224

[26] Meyer U (2014). Prenatal poly(i:C) exposure and other developmental immune activation models in rodent systems. Biol Psychiatry.

[27] Meyer U, Feldon J, Dammann O (2011). Schizophrenia and autism: both shared and disorder-specific pathogenesis via perinatal inflammation?. Pediatr Res.

[28] Meyer U, Feldon J, Yee BK (2009). A review of the fetal brain cytokine imbalance hypothesis of schizophrenia. Schizophr Bull.

[29] Meyer U, Yee BK, Feldon J (2007). The neurodevelopmental impact of prenatal infections at different times of pregnancy: the earlier the worse?. Neuroscientist.

[30] O'Loughlin EK, Pakan JMP, McDermott KW, Yilmazer-Hanke D (2014). Expression of neuropeptide Y1 receptors in the amygdala and hippocampus and anxiety-like behavior associated with Ammon's horn sclerosis following intrahippocampal kainate injection in C57BL/6J mice. Epilepsy & Behavior..

[31] Paolicelli RC, Bolasco G, Pagani F, Maggi L, Scianni M, Panzanelli P, Giustetto M, Ferreira TA, Guiducci E, Dumas L, Ragozzino D, Gross CT (2011). Synaptic pruning by microglia is necessary for normal brain development. Science.

[32] Richetto J, Calabrese F, Riva MA, Meyer U (2014). Prenatal immune activation induces maturation-dependent alterations in the prefrontal GABAergic transcriptome. Schizophr Bull.

[33] Rousset CI, Chalon S, Cantagrel S, Bodard S, Andres C, Gressens P, Saliba E (2006). Maternal exposure to LPS induces hypomyelination in the internal capsule and programmed cell death in the deep gray matter in newborn rats. Pediatr Res.

[34] Samuelsson AM, Jennische E, Hansson HA, Holmang A (2006). Prenatal exposure to interleukin-6 results in inflammatory neurodegeneration in hippocampus with NMDA/GABA(A) dysregulation and impaired spatial learning. Am J Physiol Regul Integr Comp Physiol.

[35] Schwarz JM, Bilbo SD (2011). LPS elicits a much larger and broader inflammatory response than Escherichia coli infection within the hippocampus of neonatal rats. Neurosci Lett.

[36] Smith PL, Hagberg H, Naylor AS, Mallard C (2014). Neonatal peripheral immune challenge activates microglia and inhibits neurogenesis in the developing murine hippocampus. Dev Neurosci.

[37] Stewart CR, Stuart LM, Wilkinson K, van Gils JM, Deng J, Halle A, Rayner KJ, Boyer L, Zhong R, Frazier WA, Lacy-Hulbert A, El Khoury J, Golenbock DT, Moore KJ (2010). CD36 ligands promote sterile inflammation through assembly of a Toll-like receptor 4 and 6 heterodimer. Nat Immunol.

[38] Tottenham N, Hertzig ME, Gillespie-Lynch K, Gilhooly T, Millner AJ, Casey BJ (2014). Elevated amygdala response to faces and gaze aversion in autism spectrum disorder. Soc Cogn Affect Neurosci.

[39] Vandesompele J, De Preter K, Pattyn F, Poppe B, Van Roy N, De Paepe A, Speleman F (2002). Accurate normalization of real-time quantitative RT-PCR data by geometric averaging of multiple internal control genes. Genome Biol.

[40] VanGuilder HD, Bixler GV, Brucklacher RM, Farley JA, Yan H, Warrington JP, Sonntag WE, Freeman WM (2011). Concurrent hippocampal induction of MHC II pathway components and glial activation with advanced aging is not correlated with cognitive impairment J. Neuroinflammation.

[41] Yilmazer-Hanke DM, Faber-Zuschratter H, Linke R, Schwegler H (2002). Contribution of amygdala neurons containing peptides and calcium-binding proteins to fear-potentiated startle and exploration-related anxiety in inbred Roman high- and low-avoidance rats. Eur J Neurosci.

[42] Zhan Y, Paolicelli RC, Sforazzini F, Weinhard L, Bolasco G, Pagani F, Vyssotski AL, Bifone A, Gozzi A, Ragozzino D, Gross CT (2014). Deficient neuron-microglia signaling results in impaired functional brain connectivity and social behavior. Nat Neurosci.

[43] Zhang Z, van Praag H (2015). Maternal immune activation differentially impacts mature and adult-born hippocampal neurons in male mice. Brain Behav Immun.

